# A Neural Signature of the Bias Toward Self-Focus

**DOI:** 10.1523/JNEUROSCI.0037-25.2025

**Published:** 2025-08-25

**Authors:** Danika Geisler, Meghan L. Meyer

**Affiliations:** Department of Psychology, Columbia University, New York, New York 10027

**Keywords:** default mode network, fMRI, self, social cognition

## Abstract

People are remarkably self-focused, disproportionately choosing to think about themselves relative to other topics. Self-focus can be adaptive, helping individuals fulfill their needs. It can also be maladaptive, with self-focus a risk and maintenance factor for internalizing disorders like depression. Yet, the drive to focus on the self remains to be fully characterized. We discovered a brain pattern that when spontaneously brought online during a quick mental break predicts the desire to focus on oneself just a few seconds later. In Study 1 (19 female and 13 male human subjects), we identified a default network neural signature from pr-trial activity that predicts multiple indicators of self-focus within our sample. In Study 2 (588 female and 498 male human subjects), we applied our neural signature to independent resting-state data from the Human Connectome Project. We found that individuals who score high on internalizing, a form of maladaptive self-focus, similarly move in and out of this pattern during rest, suggesting a systematic trajectory toward self-focused thought. This is the first work to “decode” the bias to focus on the self and paves the way toward stopping maladaptive self-focus in its course.

## Significance Statement

Self-help aisles in bookstores, the popularity of self-care culture, curating social media identities, and rising depression and anxiety rates are all symptoms of modern life’s emphasis on the self. Why are people so preoccupied with themselves? We discovered self-focus emerges spontaneously because of the brain pattern people enter in the default network when they have a mental break. Participants’ default network patterns in the first few seconds of rest could decode if they next chose to focus on themselves, as well as subjective and neural markers of self-focus. Moreover, individuals prone to internalizing, a maladaptive form of self-focus, systematically moved in and out of this brain pattern during rest, perhaps shaping the timing of their spontaneous, self-focused thought.

## Introduction

Writer David Foster Wallace once referred to the self as “our default setting” (Wallace, 2009). Psychological data support his view. Across cultures, people disproportionately think about themselves while mind wandering (Andrews-Hanna et al., 2010; Song and Wang, 2012; Ruby et al., 2013). They are also more likely to remember and communicate self-relevant information than information unrelated to the self (Symons and Johnson, 1997; Naaman et al., 2010). Even when people try to take the perspective of someone dissimilar to themselves, much of the time they still end up projecting their own point of view (Epley et al., 2004). While self-focus is necessary and positive in some forms—such as detecting our personal or social needs—in its most pernicious forms, self-focus is a risk and maintenance factor for internalizing disorders such as depression (Nolen-Hoeksema, 2003). Identifying what drives the bias toward self-focus would pave the way toward stopping maladaptive self-focus and preventing its negative consequences.

Yet, the processes that generate the bias toward self-focus remain to be fully determined. Insight may stem from the fact that the same parts of the brain associated with self-reflection also spontaneously engage by default whenever participants briefly pause and take a mental break. Extensive research on “the self” implicates the medial prefrontal cortex, Brodmann area 10 (MPFC/BA10), in self-reflection (Denny et al., 2012). A separate line of research consistently shows that MPFC/BA10 is a key node of the default network, known to engage “by default,” without the presence of any stimuli or experimental task (Raichle, 2015). Specifically, the default network shows stronger neural activity while passively resting relative to many tasks. Here, we asked whether the brain pattern people enter in the MPFC/BA10, and default network more generally, as soon as they have a mental break biases them toward next wanting to focus on themselves.

This possibility fits with predictive coding accounts of brain function, which broadly suggest endogenous, default brain patterns predict subsequent perception and cognition (Huang and Rao, 2011; Friston, 2018; Hutchinson and Barrett, 2019). For example, prestimulus fusiform gyrus activity predicts which of two competing visual stimuli is perceived (Hsieh et al., 2012) and prestimulus hippocampal patterns shape stimulus encoding and memory (Park and Rugg, 2010; Addante et al., 2015; Aly and Turk-Browne, 2016). To date, only one study probed prestimulus neural activity in a self-reflection task, finding that, on a trial-by-trial basis, faster responses to questions assessing beliefs about one’s traits (e.g., “Am I funny?”) are preceded by stronger MPFC/BA10 activity (Meyer and Lieberman, 2018). This finding is consistent with the present paper’s hypothesis: “defaulting” to MPFC/BA10 facilitates access to self-views. However, this still fails to identify neural processes that may set self-focus in motion. That would require default MPFC/BA10 neural responses to predict the preference to focus on the self (vs others) instead of simply quick access to self-perceptions.

In the resting-state fMRI literature, many scholars speculate that engaging MPFC/BA10, and the default network more generally, during stimulus-free rest reflects some form of self-referential processing in the scanner (Raichle, 2015; Scalabrini et al., 2022). That said, while past work has shown greater resting-state functional connectivity (i.e., timecourse correlations between brain regions) between MPFC/BA10 and other default network regions correlates with self-focus (Feurer et al., 2021; Kucyi et al., 2014), the direction of this relationship is unknown. Moreover, functional connectivity is too coarse a metric to parse the underlying mental representations considered during rest. Advances in multivariate pattern similarity analysis may help solve this issue, as the approach allows researchers to characterize the fine-grained neural patterns reflecting specific mental representations (Kriegeskorte et al., 2008). If it is possible to identify a multivariate pattern in default network regions reflecting the desire to focus on the self, this pattern could be applied to resting-state data to quantify whether endogenously engaging it predicts the bias toward self-focus.

In Study 1, we identify a default network, multivariate neural pattern that when entered during brief mental breaks biases people toward next wanting to focus on themselves. This “pre-self pattern” temporally predicts self-focus across multiple levels of analysis, including self-focused decisions, the subjective experience of self-focus, and even the presence of a separate multivariate pattern capturing active self-reflection. In Study 2, we test the generalizability and clinical relevance of the pre-self pattern. Specifically, we apply the pre-self pattern to resting-state data from the Human Connectome Project (HCP). We show that people prone to internalizing, a psychological trait characterized by maladaptive self-focus, similarly move in and out of the pre-self pattern during their resting-state scan, suggesting a systematic trajectory toward self-focused thought. Collectively, results indicate (1) we identified a neural signature in the default network that when spontaneously brought to mind during rest biases people toward next wanting to focus on themselves, and (2) this neural signature may be clinically relevant in mental health conditions characterized by atypical self-focus.

## Materials and Methods

### Study 1: identifying a neural signature that predicts self-focus

In Study 1, we sought to determine if there is a multivariate brain pattern spontaneously brought online during momentary rest that nudges the preference to think about oneself. We developed a paradigm designed to behaviorally measure the bias toward self-focus. While undergoing fMRI, participants believed they were choosing which experimental trials they would get in a separate task that immediately followed. Their decisions indeed reflected a bias toward self-focus: they disproportionately chose trials that would allow them to think about themselves (vs close and well-known others; see Results). Critical to the question of what makes us want to focus on ourselves, we assessed multivariate neural responses to each pretrial rest phase of this task and tested if it predicted choosing the self (vs others) on the immediately following trial. This approach allowed us to determine a neural pattern that can be used to “decode” the desire to think about the self before a behavioral response is made. We call this neural signature the “pre-self pattern.”

A neural signature must be generalizable—it should be able to predict self-focus in multiple ways. We next investigated if our pre-self pattern generalizes to predict self-focus in another context—a resting-state scan. First, we tested if the presence of the pre-self pattern during a resting-state scan predicts momentary reports of self-focus during that scan. Second, we tested if the presence of the pre-self pattern during the resting-state scan temporally predicted the presence of a different multivariate brain pattern capturing active self-reflection. This would suggest, even at the neural level of analysis, the pre-self pattern temporally predicts self-focus. Positive results from Study 1 would provide robust evidence that we identified a neural signature that predicts multiple characterizations of self-focus (i.e., decisions, self-report, and neural).

#### Participants

Thirty-two individuals (19 identifying as female; mean age, 22.4 years; SD = 4.5 years; 21, Caucasian; 9, Asian; 5, Multiracial; 3, Latina/Hispanic; 1, Pacific Islander; 1, Prefer Not To Say) completed our fMRI study. Participants were eligible to participate if they were safe for MRI scanning (e.g., no metal in their body, not pregnant, not claustrophobic). All participants completed informed consent in accordance with the university Institutional Review Board (IRB).

#### Procedures

##### Resting-state scan

The first fMRI run that the participants completed was an 8 min resting-state scan. The 8 min were broken up into four, 2 min sections. After each 2 min section, participants had 32 s to rate the extent to which they were thinking about themselves, others, the future, and the past. For the first six participants, this rating time was 25 s, but it was expanded to make sure participants were able to complete all four of the ratings. This was accounted for in all subsequent analyses by ensuring that all the rating TRs were regressed out in the residual image calculation stage of analysis detailed below. These ratings were made on a continuous scale with “not at all” on one end, “completely” on the other end, and “somewhat” in the middle of the scale. Participants utilized a trackball mouse to make their selection, and a value between 1 and 5, to the 14th decimal point, was recorded for each of the categories (self, other, future, and past).

##### The choice task: capturing the bias toward self-focus

**Figure 1.**
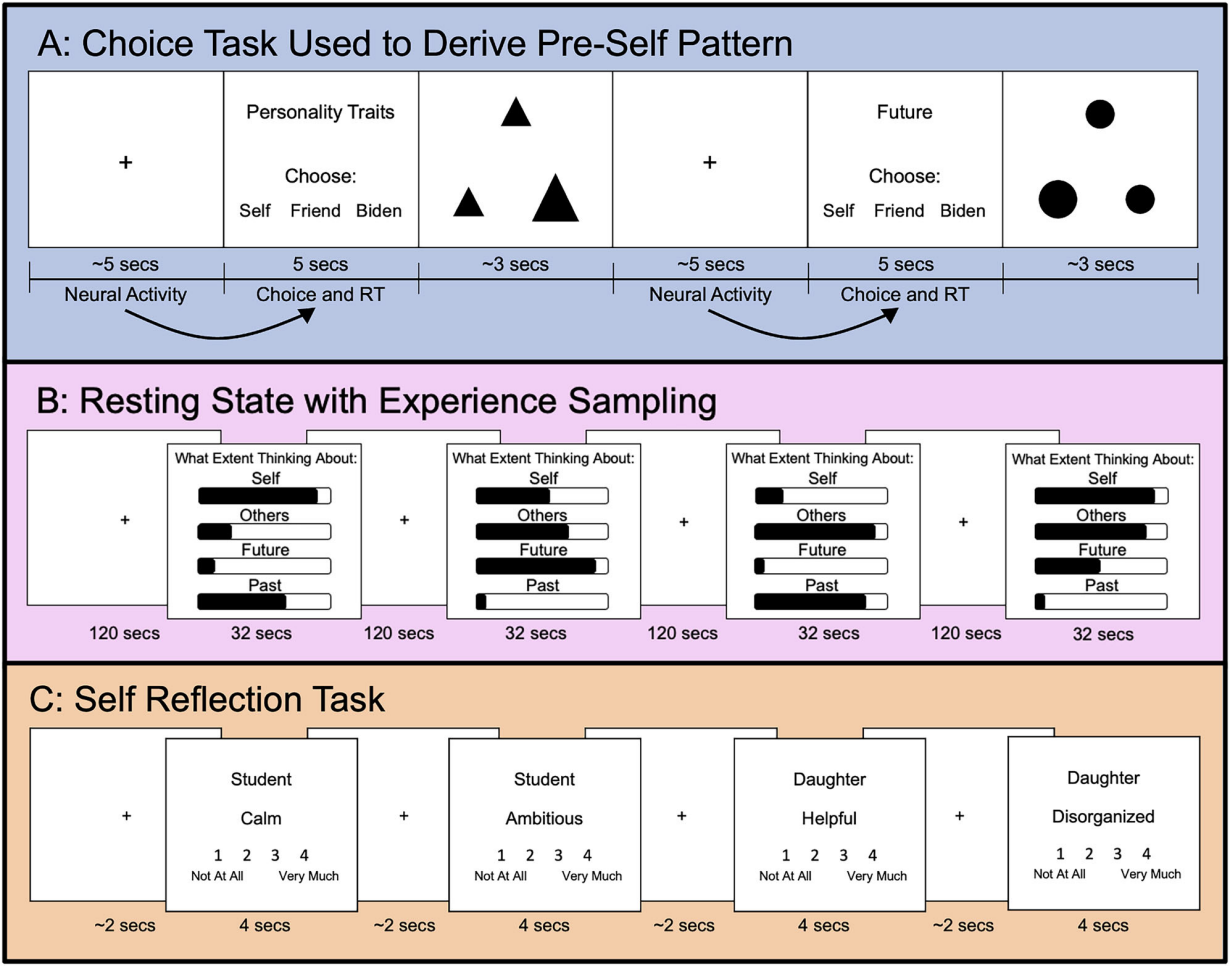
***A***, The choice task (used to derive the pre-self pattern) included three parts that repeated. First was a pretrial jittered rest period (2.5–6 s; mean, 4.5 s); second was the choice activity where participants chose who (themselves, a designated friend, or Biden) they wanted to think about in a later task (5 s); third was a shape-matching task aimed at moving participants' minds off their last choice. These three parts repeated 54 times in run 1 and 54 times in run 2 for a total of 108 trials. Our analysis examined neural activity during the pretrial jittered rest in relation to the subsequent task choice and response time. ***B***, The first fMRI run that the participants completed was an 8-min-long resting-state scan. The 8 min were broken up into four, 2-min-long sections. After each 2 min section, participants had 32 s to rate the extent to which they were thinking about themselves, others, the future, and the past. These ratings were made on a continuous scale with “not at all’ on one end, “completely” on the other end, and “somewhat” in the middle of the scale. ***C***, The final task participants completed was a typical self-reflection task. In this task participants were instructed to rate how well an adjective describes their personality in different social roles (Friend, Student, Significant Other, Son/Daughter, and Worker) on a scale from 1 to 4 using button boxes. We used the Big 5 list of 100 adjectives (Goldberg, 1993a). The rating trials were 4 s long and the jittered rest was 1–3 s, with mean of 2 s. There were two runs of 101 trials each, with a total of 202 trials.

For the main “choice” task, designed to measure the bias toward self-focus, participants were led to believe that they were choosing the trial types they would receive in a separate task that would follow. They were told this follow-up task involved two 9 min scans in which they would reflect for 10 s on every trial type that they had just selected. In actuality, after completing this choice task, all participants proceeded to get the same task, described below. The choice task started with a pretrial jittered rest period (2.5–6 s; mean, 4.5 s), followed by the target choice activity where participants choose who (themselves), a close other (i.e., a self-nominated friend), or a well-known other (i.e., President Biden) they wanted to think about in the later task (5 s). This design is an adaptation of previous fMRI paradigms used to assess self-reflection (Kelley et al., 2002; Zhu et al., 2007; Jenkins and Mitchell, 2011; Meyer and Lieberman, 2018). To make the task more engaging, and to confirm default MPFC/BA10 brain patterns predict self-focus broadly, as opposed to self-focus specifically along a certain dimension(s), we included six different categorical dimensions such that participants made choices about who to think about along the following dimensions: social roles (e.g., being a daughter), preferences, physical traits, personality traits, future, and past. Participants made a total of 108 choices (split into two runs each lasting 11 min and 40 s), evenly distributed between the dimensions with 18 choices per dimension. After each decision of which target they would think about in the later scanner task, participants completed an attention reorienting activity in which they picked which of the two bottom shapes matched the top shape (2–4.5 s; mean, 3 s). This attention reorienting activity served as a “mental palate cleanse,” to get participants’ minds off of their last target choice before the next jittered rest. Participants were instructed not to actively reflect on the topic and target that they selected, but instead to make the decision that first came to mind, and next focus on the shape-matching activity. See Figure 1*A* for a schematic of the task.

Note that it was made clear to participants that for every single “choice” they made in the primary task (108 trials), in the following task they would get a corresponding trial that reflected that choice. For example, if on a “choice” trial they were given the choice: “Traits: Self, Biden, Friend” and they chose “Self,” then they believed that for one of 108 trials on their next task, they would get a personality trait question about themselves (“Are you kind? Yes or No?”). This means every decision they made in the primary decision task was consequential for their next task. We did not explain why they got to choose their next trials; it was just part of the experiment.

##### Self-reflection task

The last fMRI task that participants completed for the study was an active self-reflection task. In this task, participants rated how well an adjective described their personality in different social roles (Friend, Student, Significant Other, Son/Daughter, and Worker) on a scale from 1 to 4 using button boxes. We used the Big 5 list of 100 adjectives (Goldberg, 1993b). These are adjectives evenly split among the Big 5 (agreeableness, conscientiousness, intellect, emotional stability, and surgency) as well as positive and negative valence. The social role participants reported on shifted every 10 adjectives. We used a change in text color to ensure participants noted this change. The number of adjectives from each of the Big 5, as well as the positive and negative valence, were balanced across the five roles. The rating trials were 4 s long and the jittered rest was 1–3 s, with mean of 2 s. There were two runs of 101 trials each, with a total of 202 trials. We used this task here to generate, for each subject, a neural pattern of their active self-reflection. Other analyses can be run with this task to answer separate theoretical questions but are outside of the scope of this report and will not be examined here.

#### Behavioral analysis

For the choice task designed to detect the bias toward self-focus, we first conducted a chi-square test of independence to test whether the number of selections varied for the three targets (self vs friend vs Biden). Then, because the test was significant, we followed up with paired-sample chi-square tests of independence to confirm the interaction is driven by more decisions for the self. These chi-square tests of independence were performed using the R package *stats* (R Core Team, 2022) to compare choice of subject (self, friend, or Biden). An analysis of variance (ANOVA) tested whether RT varied across the target factor (self vs friend vs Biden). Next, because the results were significant, linear mixed models using the R package *lme4* were constructed to assess how choice of target (self, friend, or Biden) affected the response time (Bates et al., 2015).

#### fMRI collection

Brain imaging took place on a Siemens Prisma 3T scanner. Functional runs were acquired using a T2*-weighted echoplanar imaging sequence [2.5 mm voxels; repetition time (TR), 1,000 ms; time to echo (TE), 30 ms; 2.5 mm slice thickness; field of view (FOV), 24 cm; matrix, 96; flip angle, 59; and simultaneous multislice, 4]. A T2-weighted structural image was acquired coplanar with the functional images (0.9 mm voxels; TR, 2,300 ms; TE, 2.32 ms; 0.9 mm slice thickness; FOV, 24 cm; matrix, 256·256; and flip angle, 8) for the purpose of aligning functional data to brain structure during preprocessing. For the fMRI tasks, sequence optimization was obtained using optimizeXGUI in Matlab (Spunt, 2016).

#### fMRI preprocessing

For the fMRI dataset we collected, results included in this manuscript come from preprocessing performed using fMRIPrep 20.2.2 (Esteban et al., 2019; RRID:SCR_016216). Per recommendations from the software developers, we report the exact text generated from the boilerplate in Supplementary Materials (S1 Text). Briefly here, preprocessing included skull stripping, motion correction, slice time correction, smoothing, registration, and normalization.

#### Residual image calculation

For the main “choice task” designed to measure the bias toward self-focus, in order to examine neural activity during pretrial jittered rest that is not contaminated by neural responses during the target choice activity or shape-matching activity, we first regressed out the effects of the target choice activity and shape-matching activity as well as the effects of motion. This step is in line with prior research examining pretrial neural responses (Meyer and Lieberman, 2018). All analyses of the pretrial jitter period were run on the residual images saved from those models. Specifically, residual images were calculated using Python’s *nltools* (Chang et al., 2023) by modeling the choice task and shape-matching task convolved with the canonical hemodynamic response function in a general linear model. This model included nuisance regressors for the six motion parameters (*x*, *y*, and *z* directions and roll, pitch, and yaw rotations), each motion parameter’s derivative and square of the derivative, linear drift, and run constants. We additionally regressed out TRs in nonsteady state and TRs that exhibited spikes of motion found from global signal outliers and outliers derived from frame differencing (each 3 SDs). We used a smoothing kernel of 6 on the residual images that were used for the parametric modulation analysis but no smoothing kernel on the residual images used for the multivoxel pattern analysis (MVPA) analysis given that they require the fine-grained, voxel-level detail.

Consistent with prior resting-state research (Meyer et al., 2019), we also calculated residual images for the resting-state scan. To examine neural activity during rest that is not biased by neural activity during the self-report rating activity, we first regressed out the effects of the self-report rating activity as well as the effects of motion. All analysis of the resting-state periods were run on the residual images saved from those models. Specifically, residual images were calculated using Python's *nltools* (Chang et al., 2023) by modeling the rating task convolved with the canonical hemodynamic response function in a general linear model. This model included nuisance regressors for the six motion parameters (*x*, *y*, and *z* directions and roll, pitch, and yaw rotations), each motion parameter's derivative and square of the derivative, linear drift, and run constants. We additionally regressed out TRs in nonsteady state and TRs that exhibited spikes of motion found from global signal outliers and outliers derived from frame differencing (each 3 SDs). Finally, we used a smoothing kernel of 6.

#### Multivoxel pattern analysis

##### Pre-self pattern generation

To determine if we can “decode” the bias toward self-focus from pretrial rest, for the choice task, we performed MVPA on a whole-brain activation map masked (separately) by MPFC/BA10, the three subsystems of the default network (the core subsystem, dMPFC subsystem, and MTL subsystem) as identified by Yeo et al. (2011), as well as an unmasked whole-brain activation map. Specifically, the pre-self pattern is the classifier weights map generated by the multivoxel pattern classification analysis performed with all subjects' activation maps. The validity of the classifier was tested with a sixfold balanced cross-validation procedure.

The masks were chosen because we were interested in assessing MPFC/BA10’s roles in particular and the default network’s role more broadly in prompting us toward self-focus. The unmasked whole brain was also analyzed because its classification accuracy acts as a useful comparison. If a mask (e.g., core subsystem) has a higher classification accuracy than the whole brain, this suggests the whole brain's classification power is disrupted by the noise created by areas outside of the mask. We again assessed classification of self versus other, where friend and Biden were combined into the other category. We focused on this self versus other classification so that there was a near equal number of trials in each group, which helps ensure an unbiased classification result.

To generate the input for the classifier, we used *nltools* (Chang et al., 2023) to create a first-level model (performed on participants’ residual images) that included three regressors reflecting the jittered rest period before each target choice: a rest before self-choice regressor, a rest before friend-choice regressor, and a rest before Biden-choice regressor. A baseline Beta of self and other (combining friend and Biden) was then extracted and utilized as the input to our classifier. In other words, for each subject, there was a different Beta value map for rest periods: (1) preceding self-focused decisions and (2) preceding other-focused decisions. We trained a linear support vector machine (SVM) to discriminate a subsequent choice of self (coded as 1 in the classification) versus other (coded as −1 in the classification). In the statistical learning literature, there are many types of classification algorithms, but they generally perform very similarly on problems such as the one we pursued here (Hastie et al., 2008). SVM algorithms such as the one we used in this study are the most widely used algorithm for two-choice classification and are robust and reasonably stable in the presence of noisy features.

We computed prediction performance using a sixfold balanced cross-validation procedure (Cohen et al., 2010; Izuma et al., 2018). We subdivided the data into six separate folds (5−6 participants in each group) and used all of the data except for one fold to train the model and then tested the model using the left-out fold. We then iterated over this process for every possible fold. We chose the *k*-folds balanced cross-validation approach, instead of a leave-one-out subject approach, because it is less susceptible to outlier participants (Vu et al., 2022).

The sixfold balanced cross-validation procedure was completed for each of our five neural maps (MPFC/BA10, whole brain, default network core subsystem, dmPFC subsystem, and MTL subsystem), and an average classification accuracy was calculated for each. A follow-up analysis was then done following the same steps with all of the ROIs included in Yeo’s (2011) default network core subsystem (right middle temporal gyrus, right superior frontal gyrus, left middle frontal gyrus with left superior frontal gyrus, left angular gyrus, right angular gyrus with right middle temporal gyrus, and PCC) except for MPFC/BA10 as that ROI was already analyzed. Another follow-up analysis was also done utilizing the same methods with four ROIs, (1) default network core subsystem minus MPFC, (2) default network core subsystem minus the PCC, (3) default network core subsystem minus MPFC/BA10 and PCC, and (4) MPFC/BA10 and PCC combined. We ran statistical tests on a total of 15 classifier models over the course of this analysis. To account for issues with multiple comparisons, statistical results are only reported as significant if they have *p* < 0.05/15 or 0.003.

To test the statistical significance of these results, we generated null distributions for each ROI using 10,000 permutations of a sixfold SVM classification analysis. First, for each subject we relabeled their self and other neural images randomly as self or other. Then we subdivided the data into six separate folds (5–6 participants in each group) and used all of the data except for one fold to train the model and then tested the model using the left-out fold. We then iterated over this process for every fold. This was repeated 10,000 times, and the average classification accuracy of each permutation was used to create a null distribution. Our average classification accuracy result was then compared with this null distribution using nltools (Chang et al., 2023). This process was completed for all 15 of the neural maps mentioned above (whole brain, default network core subsystem, dmPFC subsystem, MTL subsystem, right middle temporal gyrus, right superior frontal gyrus, left middle frontal gyrus with left superior frontal gyrus, left angular gyrus, right angular gyrus with right middle temporal gyrus, PCC, MPFC/ACC, default network core subsystem minus MPFC, default network core subsystem minus the PC, default network core subsystem minus MPFC/BA10 and PCC, and MPFC/BA10 and PCC combined). Because results from this analysis implicated the default network core subsystem, all subsequent analyses described below move forward with the default network core subsystem as the primary mask and follow-up analyses testing for specificity to this subsystem are run with the other Yeo default network subsystems/ROIs (Yeo et al., 2011).

##### Testing whether pre-self pattern instatement during the resting-state scan predicts subjective reports of self-focus

The analysis described above answers whether multivariate patterns in the default network can decode self-focused choices. Next, once we characterized the pre-self pattern from the choice task, we could apply it to our resting-state data to answer our next question. Specifically, we asked if the stronger presence of the pre-self pattern during rest predicted participants’ subjective ratings of self-focus during their resting-state scan. We performed an instatement analysis—a TR-to-TR pattern matching analysis with the classifier pattern generated by the MVPA of the default network core subsystem. Each TR of the 8 min of rest was masked by the Yeo default network core subsystem ROI and then correlated with the default network core subsystem pre-self pattern. These correlation values were then averaged for the 2 min of rest before each rating and *z*-scored. Linear mixed models using the R package *lme4* were constructed to assess how self-report of thought content (self, other, future, and past) as well as the section of rest affected the mean correlation (Bates et al., 2015). To ensure that the regressors were not introducing collinearity to the model, we ran tolerance and variance inflation factors. All VIF results were well below the threshold of 5 (Kutner et al., 2005; Self, 1.2; Other, 1.1; Future, 1.3; Past, 1.1; and Section, 1.1), and all tolerance values were over 75% (Self, 82; Other, 90; Future, 77; Past, 88; and Section, 93). To follow up on this, analyses were also run with the MVPA pre-self classification patterns generated with the dmPFC subsystem, MTL subsystem, MFPC, PCC, and whole brain.

##### Self-reflection pattern generation

Next, we turned to our third question: does the presence of the pre-self pattern during the resting-state scan even predict the presence of an active self-reflection neural pattern a few seconds later? This would again be consistent with the idea that the pre-self pattern temporally predicts future self-focus, here “neural self-focus.” To test whether our pre-self pattern temporally predicts the presence of active self-reflection during a resting-state scan, we needed to create a neural pattern reflecting active self-reflection and subsequently examine the temporal relationship between pre-self pattern instatement and instatement of self-reflection neural activity during the resting-state scan. To this end, we generated neural patterns for each subject using the self-reflection task. Specifically, images were calculated by modeling the self-reflection task convolved with the canonical hemodynamic response function in a general linear model. This model included nuisance regressors for the six motion parameters (*x*, *y*, and *z* directions and roll, pitch, and yaw rotations), each motion parameter's derivative and square of the derivative, linear drift, and run constants. We additionally regressed out TRs in nonsteady state and TRs that exhibited spikes of motion found from global signal outliers and outliers derived from frame differencing (each 3 SDs). Finally, we used a smoothing kernel of 6. We created a contrast of self-reflection compared with baseline and saved the resulting image (i.e., “activation map”) for each subject to use in the subsequent instatement analysis.

##### Testing the temporal relationships between pre-self and self-reflection pattern instatement during a resting-state scan

Given that we now had the pre-self classification pattern and, for each subject, an active self-reflection neural pattern (see above, Self-reflection pattern generation), we could test whether the presence of the pre-self pattern temporally predicted the presence of an active self-reflection neural pattern. We performed an instatement analysis—a TR-to-TR pattern matching analysis—with the classification pattern generated by the MVPA of the default network core subsystem and with the active self-reflection neural pattern generated with the self-reflection task. First, we masked the active self-reflection pattern of each subject by the Yeo default network core subsystem ROI (Yeo et al., 2011). Then each TR of the 8 min of rest was masked by the Yeo default network core subsystem ROI and correlated with the default network core subsystem pre-self pattern as well as the masked version of the active self-reflection pattern. Linear mixed models using the R package *lme4* (Bates et al., 2015) were constructed to assess if pre-self pattern correlation strength predicted active self-reflection pattern correlation strength 0–20 TRs later (see Fig. 5*A* for a visualization of the approach). We chose to use this data-driven approach with multiple TR lag times to avoid arbitrarily selecting a time delay as well as to better understand the dynamic temporal relationship between the presence of the “pre-self” pattern and active self-reflection pattern.

Analyses are performed on fisher *z*-transformed correlation values. We tested the correlation of the two neural patterns for each subject and did find small (*r* = 0.08 to −0.03). We therefore ran the linear mixed models with 0–20 TR delays: (1) with the strength (i.e., correlation) of the pre-self pattern predicting the strength of the active self-reflection pattern X TRs later and (2) with the strength (i.e., correlation) of the active self-reflection pattern predicting the strength of the pre-self pattern X TRs later. This enabled us to make sure that the correlation of the two patterns was not the cause of significant temporal results. To account for the number of statistical tests performed (41), we used a Bonferroni-corrected significance value of *p* < 0.00122 (i.e., 0.05 × 41). Significant results are reported here; see Study 1 Table S1 for a full list of the results for each TR lag.

### Study 2: testing implications for internalizing and out-of-sample testing

The results from Study 1 suggest that we can decode the bias toward self-focus in our own dataset (see Results). The pre-self pattern predicts self-focused decisions, subjective self-focus during rest, and the presence of active self-reflection neural patterns a few seconds later.

Self-focused thought is implicated in mental health conditions, particularly internalizing disorders such as anxiety and depression (Watkins, 2008; du Pont et al., 2018; Watkins and Roberts, 2020; Baez et al., 2023). Could the pre-self pattern be used to predict internalizing scores in an entirely separate sample of participants? If so, this would have great utility: it would suggest we derived a neural marker that, down the line, could be used to identify a person’s vulnerability to internalizing conditions. In a first step toward this goal, we took the pre-self pattern, created in our 32 subject dataset, and applied it to a larger dataset to show that the pattern translates not just across tasks but also across datasets and subjects. In Study 2, we tested whether our pre-self pattern predicts outcomes related to maladaptive self-focus in data from the Human Connectome Project. The Human Connectome Project dataset includes a resting-state scan and internalizing score for each participant, which reflects symptoms like anxiety and depression that are related to self-focus.

We arbitrated between two ways the presence of the pre-self pattern may predict internalizing. The first possibility is a matter of “degree”: an overall greater amount of pre-self pattern instatement during a resting-state scan may predict internalizing. Trait internalizing is considered a stable tendency to engage in the self-focused thought characteristic of internalizing disorders (Eaton et al., 2011). Consistent with the degree possibility, individuals with (vs without) internalizing disorders subjectively report being more strongly focused on themselves relative to other topics (Andrews-Hanna et al., 2013) and are more likely to reference their personal experience in laboratory tasks (Ingram and Smith, 1984) and in naturalistic settings, such as everyday conversation (Jacobson and Anderson, 1982; Mehl, 2006). It is possible that these behavioral outcomes indicating greater self-focus occur, at least in part, because individuals prone to internalizing have a stronger presence of their pre-self pattern. That said, the “degree” possibility is complicated by the fact that while internalizing is linked to the default network, the literature is mixed with respect to whether internalizing corresponds with greater or weaker default network engagement (Berman et al., 2011; Modi et al., 2015; Javaheripour et al., 2021; Tozzi et al., 2021). While internalizing relates to self-focus, it is unclear that this reflects overall stronger engagement of the pre-self pattern.

The second possibility is a matter of “fashion,” or the way in which trait internalizers dynamically move in and out of the pre-self pattern over time. People similar to one another on a psychological dimension show similar time courses of neural engagement, not only in response to stimuli, but also during resting-state scans (Finn et al., 2018; Iyer et al., 2024). Prior work has also correlated multivariate templates indicative of task behavior (i.e., behavioral arousal) with each temporal acquisition of fMRI resting-state data (i.e., “repetition time” or, TR) to show (1) participants systematically transition in and out of an arousal state during rest and (2) the transition timing corresponds with psychological experience during rest (Chang et al., 2016). These observations, in conjunction with prior work showing internalizers have a strong tendency to spontaneously engage in self-focused thought (Andrews-Hanna et al., 2013), suggest that trait internalizers may have a systematic timing to the presence of their pre-self pattern during rest. The “fashion” possibility also fits with research on rumination—repetitive and recurrent negative thinking about oneself—which is thought to play a key role in internalizing disorders (du Pont et al., 2018; Watkins and Roberts, 2020; Baez et al., 2023). Mirroring the flow of ruminative thought, there may be a temporal structure to the presence of the pre-self pattern among internalizers. If either the “degree” or “fashion” possibility is correct, it would provide key evidence that (1) we identified a neural signature that meaningfully predicts outcomes related to self-focus in independent datasets and (2) that the neural signature is clinically relevant.

#### Human Connectome Project participants

We applied our pre-self pattern to data from the first resting-state scan of the Human Connectome Project, hereafter referred to as the Human Connectome Project dataset. The dataset is openly accessible and consists of a large sample of neurotypical individuals. Data from the Human Connectome Project are publicly available in the online Human Connectome Project repository (https://db.humanconnectome.org/; fMRI data are in the subfolders rfMRI_REST1_RL; behavioral data are in the Restricted Data file). We utilized neural and behavioral data from 1,086 individuals (age 22–37 years; mean age 28.8; 588 female and 498 male; 817, White; 63, Asian/Nat. Hawaiian/Other Pacific Is.; 158,Black or African Am.; 2, Am. Indian/Alaskan Nat.; 28, More than one; 18, Unknown or Not Reported; 979, Not Hispanic/Latino; 94, Hispanic/Latino; 13, Unknown or Not Reported). Trait internalizing was measured with the Adult Self Report (ASR) from the Achenbach System of Empirically Based Assessment (ASEBA; Achenbach et al., 2017). Follow-up analysis also used the three subscales within the internalizing measure—(1) Anxiety and Depression, (2) Withdrawal, and (3) Somatic Complaints.

#### fMRI collection

The resting-state scan that we used in our analysis was 14 min 33 s long and was the first functional scan done on participants’ first day in the lab. The fMRI data were acquired using a 3T Skyra scanner with 2 mm isotropic voxels and a TR of 0.72 s. Each run comprised 1200 scan volumes, and there was a single run for each participant. We used minimally preprocessed voxelwise fMRI data.

#### Residual image calculation

Consistent with Study 1's data analysis pipeline and prior resting-state research (Meyer et al., 2019), we also calculated residual images for the Human Connectome Project resting-state scan. Residual images were calculated with a general linear model that included nuisance regressors for the six motion parameters (*x*, *y*, and *z* directions and roll, pitch, and yaw rotations), each motion parameter's derivative and square of the derivative, and linear drift. We additionally regressed out TRs in nonsteady state and TRs that exhibited spikes of motion found from global signal outliers and outliers derived from frame differencing (each 3 SDs). Finally, we used a smoothing kernel of 6. All analyses of the Human Connectome Project resting-state scan were run on the residual images saved from those models.

#### Linking pre-self pattern instatement and internalizing in the Human Connectome Project dataset

For each Human Connectome participant, we first carried out an instatement analysis in which we performed a TR-to-TR pattern matching analysis with our pre-self pattern—the classifier pattern generated by the MVPA of the default network core subsystem. Each TR of the 14.5 min of rest was masked by the Yeo (2011) default network core subsystem ROI and then correlated with the default network core subsystem pre-self pattern. This created a time course for each subject where each data point indicated the extent to which the participant's neural activity matched our pre-self pattern at that particular time point during rest (Fig. 6*A*).

##### Average pre-self pattern instatement during rest and internalizing

We arbitrated between two ways in which instating the pre-self pattern may relate to internalizing. The first possibility is that an overall greater degree of pre-self pattern instatement predicts internalizing, given that individuals higher on this trait dimension report an overall greater amount of self-focus. To test the first possibility, after we performed the instatement analysis detailed above, we averaged pre-self pattern instatement values across all TRs for each participant. We then created a linear model in R with the *stats* package (R Core Team, 2022) to determine if participant’s internalizing scores significantly predicted their average pre-self pattern instatement during rest.

##### Intersubject representational similarity analysis

The second possibility is that individuals who score high on internalizing show a similar time course to the presence of their pre-self pattern, moving in and out of the pre-self pattern similarly over the course of the resting-state scan. To test this prediction explicitly, we used an intersubject representational similarity analysis (IS-RSA; Finn et al., 2020) to test an “Anna Karenina” model. Statistically, Anna Karenina models test if “all high (or low) scorers are alike; each low (or high) scorer is different in his or her own way,” with the researcher specifying whether they predict similarity among high or low scorers (Chang et al., 2020; Finn et al., 2020). In our case, we specified the model to test if all high internalizers move in and out of the pre-self pattern alike, while low internalizers move in and out of the pre-self pattern in their own unique ways.

Following suggestions from Finn et al. (Finn et al., 2020), we modeled subject similarity based on participants’ absolute position on the internalizing scale by computing the mean internalizing rank [i.e., (rank(*i*) + rank(*j*)) / 2]. This approach is the appropriate choice for Anna Karenina models, as opposed to Euclidean distance or other relative distance measures (which are used in nearest neighbor models), because Euclidean distance between subjects would be agnostic to where on the internalizing continuum participants fall. We hypothesized that high-internalizing individuals exhibit greater similarity among themselves, while low-internalizing individuals are less similar to both high-internalizing individuals and low-internalizing individuals. Therefore, we modeled subject similarity via mean internalizing rank, which captures this relationship. See Figure 6*B* for a depiction of the Anna Karenina model.

IS-RSA requires computing two subject-by-subject matrices (here, one matrix for subjects' internalizing scores and one matrix for subjects' pre-self pattern instatement time courses) and statistically comparing them to one another. To create the internalizing score matrix, subjects’ internalizing scores were turned into ranks, making low-internalizing subjects ranked low- and high-internalizing subjects ranked high (range of ranks = 0–1,085 for *N* = 1,086). We then computed the mean of these ranks for every subject pair to generate our 1,086*1,086 intersubject mean internalizing matrix. Next, we Pearson correlated each subject's pre-self pattern instatement time course with every other subjects’ pre-self pattern instatement time course to populate the neural representational similarity matrix. This instatement time course similarity matrix was organized as a function of participant's internalizing scores, so that participants higher on internalizing appeared at the top and those lower on internalizing appeared on the bottom (Fig. 6*A*,*B*).

Next, to test our hypothesized link between high internalizing and similar pre-self pattern time courses during rest, we Spearman correlated our intersubject internalizing and instatement time course similarity matrices (specifically, correlating only the lower triangles of these symmetric matrices). We used Spearman correlations, as is the protocol in the representational similarity analysis literature (Kriegeskorte et al., 2008), because the increase in pre-self pattern instatement time course similarity may not be linear to either the increase in the internalizing of a subject pair or the increase in the internalizing similarity of a subject pair.

To test our model's statistical significance, we needed to account for each subject appearing in the model multiple times—as we compared every subject to every other subject indicating each subject appears *N*−1 (1,085) times in our model. To account for this nonindependence in our data, we tested our model with a nonparametric, Mantel permutation test, as has been done in previous research (Iyer et al., 2024). Specifically, we randomly shuffled the identity associated with subjects' instatement time courses [e.g., each subject's (intact) instatement time course was relabeled with a different subject's identity] 100,000 times, each time correlating the resulting simulated instatement time course similarity matrix with our unshuffled Anna Karenina calculated internalizing matrix. This created a null distribution of IS-RSA correlation values. We then quantified the probability that our results were produced by chance by computing the proportion of times our simulated null correlation value exceeded our observed model data correlation. Finally, to determine statistical significance, we compared this probability to a significance threshold of alpha = 0.05. As a follow-up, we also performed IS-RSA Anna Karenina analyses separately for each of the three subscales within the internalizing measure—(1) Anxiety and Depression, (2) Withdrawal, and (3) Somatic Complaints. We replicated our previous methodology three times, substituting each subscale measure for the overall internalizing measure.

## Results

### Study 1: identifying a neural signature that predicts self-focus

#### When given the choice, people prefer to think about themselves over others

**Figure 2.**
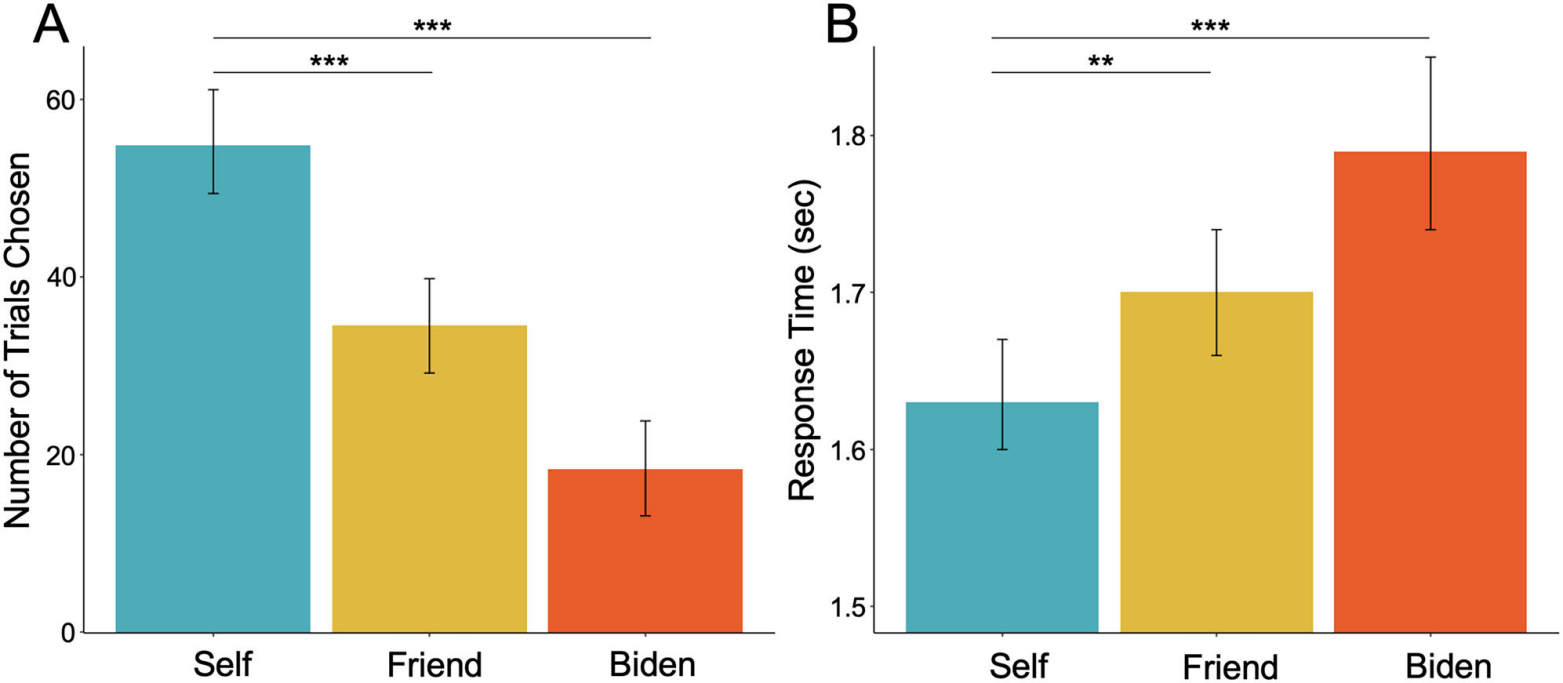
***A***, Participants choose to think about themselves significantly more than their self-nominated friend [*χ*^2^ (1, *N* = 2,823) = 114.69, *p* < 0.001] or Biden [*χ*^2^ (1, *N* = 2,320) = 495.34, *p* < 0.001]. ***B***, Participants are faster in their decisions to think about themselves in comparison with a friend (*β* = 0.11, standardized *β* = 0.02, *t*_(3429)_ = 4.68, *p* = 0.003) and Biden (*β* = 0.08, standardized *β* = 0.03, *t*_(3435)_ = 2.93, *p* < 0.001). *** indicates *p* < 0.001; ** indicates *p* < 0.005.

In our primary fMRI task, participants believed they were choosing trials that would appear in their subsequent fMRI task. Specifically, they choose if they would later like to think about themselves, a close other (i.e., nominated friend), or a well-known other (i.e., President Biden) across multiple dimensions assessed separately (i.e., personality and physical traits; social roles; preferences; past and future), with a total of 108 choices made (Fig. 1*A*). Participants' decisions indicated a strong predisposition to default toward self-focus (Fig. 2). A chi-square test of independence demonstrated a main effect of choice on the number of trials selected [*χ*^2^ (2, *N* = 3,447) = 500.71, *p* < 0.001]. Follow-up, paired-sample chi-square tests of independence showed participants choose to think about themselves significantly more than a designated friend [*χ*^2^ (1, *N* = 2,823) = 114.69, *p* < 0.001] and Biden [*χ*^2^ (1, *N* = 2,320) = 495.34, *p* < 0.001]. Using an ANOVA, we also found a main effect of choice on response time (*F*_(2,3431.3)_ = 11.97, *p* < 0.001). Follow-up linear mixed models showed that participants were faster to make decisions to think about themselves in comparison with a friend (*β* = 0.11, standardized *β* = 0.02, *t*_(3429)_ = 4.68, *p* = 0.003) and Biden (*β* = 0.08, standardized *β* = 0.03, *t*_(3435)_ = 2.93, *p* < 0.001). Notably, participants were also more likely to choose to think about their friend than Biden [*χ*^2^ (1, *N* = 1,751) = 144.49, *p* < 0.001] and were faster to choose their friend over Biden (*β* = 0.08, standardized *β* = 0.03, *t*_(3435)_ = 2.93, and *p* < 0.001). Supplementary Materials (Text S1) present detailed results from three follow-up analyses that further confirm the robustness of the bias toward self-focus. Briefly here, the bias to choose the self over others was not due to certain dimensions (e.g., choosing to think about one's personality traits vs future), appeared consistently across the experimental task, and was not affected by the length of the rest preceding the decision.

#### A neural signature that predicts self-focus: evidence from the brief, pretrial rest in the choice task

##### Univariate MPFC/BA10 activity during pretrial rest parametrically modulates how quickly participants choose to think about the self (vs others)

The primary goal of the choice task was to derive a neural signature that precedes and decodes self-focus. Meeting that goal requires using a multivariate approach to classify participants' decisions. However, before meeting that primary goal, we first wanted to assess if we conceptually replicate the single, previous study investigating the role of prestimulus responses on self-referential processing (Meyer and Lieberman, 2018). To date, one study examined prestimulus neural activity in a self-reflection task and discovered that, on a trial-by-trial basis, stronger MPFC/BA10 activity during prestimulus fixation predicts faster responses to immediately following questions assessing beliefs about one’s traits (e.g., “Am I funny?” yes/no; Meyer and Lieberman, 2018). This finding complements the present paper’s hypothesis: it shows that default MPFC/BA10 activity may help people quickly access self-knowledge. However, speed is not the same thing as desire. The prior study does not speak to whether prestimulus, default brain patterns predict wanting to focus on the self (i.e., the bias toward self-focus). We first asked whether we conceptually replicate this prior work, testing if the average amount of pretrial activity (i.e., univariate activity) relates to task performance on a given trial—specifically, faster reaction time to self versus other choices.

Consistent with the prior work, we performed a parametric modulation analysis in which univariate neural activity during pretrial rest was modulated—on a trial-by-trial basis—by the speed and target choice of the next trial. Note that these and all subsequent analyses are run on the residual images from the task activation models to ensure pretrial rest activity is not contaminated by task-evoked effects. Given that the previous work found the magnitude of MPFC/BA10 activity preferentially facilitates the speed with which participants answer questions about themselves, we probed the parametric modulation analysis here in an MPFC/BA10 region of interest (ROI) predefined by Yeo et al. (2011). Consistent with the prior work, faster decisions to choose the self (vs friend and Biden) corresponded with greater mean activity in MPFC/BA10 during the previous pretrial rest period (*t*_(31)_ = −2.20, *p* = 0.036, Cohen's *d* = −0.39). No additional clusters emerged in follow-up whole-brain parametric modulation analyses searching for any clusters whose greater activation during pretrial rest predicted faster decisions to think about: (1) self versus other (friend and Biden), (2) friend versus self, (3) Biden versus self, or (4) friend versus Biden. Additionally, a follow-up, whole-brain parcellation analysis with 85 ROIs defined by Yeo et al. (2011) again indicated no other regions showed a significant parametric relationship with these four contrasts (Bonferroni corrected *p*'s > 0.60). Conceptually replicating prior work, the results show that faster choices to think about the self (vs others) are preceded by greater mean MPFC/BA10 activity. For more details about these analyses, see the Parametric Modulation Methods in the Supplementary Materials (Text S1).

##### Multivariate neural patterns in the core default network subsystem during pretrial rest predict decisions to think about the self

Next, we returned to our primary goal—to test whether we could use multivariate neural patterns during pretrial rest to “decode” if participants subsequently wanted to think about themselves. In the decoding analysis presented here, we used the decision of target in the choice task as the outcome variable to determine if neural patterns during pretrial rest predict the subsequent choice to think about the self. Specifically, using MVPA on the pretrial rest, we trained a linear SVM classifier to differentiate between subsequent self-choices versus other choices (friend and Biden). Friend and Biden choices were combined so there was a roughly equal number of self versus other trial types, which helps ensure the analysis is unbiased.

To generate the input for the classifier, we created a first-level model. This was performed on participants’ residual images from the task activation model, to ensure pretrial rest activity is not contaminated by task-evoked effects. The first-level model comprised three regressors for the rest period before each trial. These regressors modeled pretrial rest before trials in which the participant made the choice to be (1) self-focused or to be (2) other-focused. A baseline Beta value of each of the decision types—self and other—was then extracted and utilized as the input to our classifier. In other words, for each subject, there was a different Beta value map for rest periods (1) preceding self-focused decisions and (2) preceding other-focused decisions.

We first performed the analysis and validation specifically within the Yeo et al. (2011) MPFC/BA10 ROI. We computed prediction performance using the sixfold balanced cross-validation procedure (Cohen et al., 2010; Izuma et al., 2018). Specifically, we subdivided the data into six separate folds (5–6 participants in each group) and used all of the data except for one fold to train the model and then tested the model using the left-out fold. We then iterated over this process for each fold, and an average classification accuracy was calculated. A null distribution was computed using 10,000 permutations of sixfold randomized SVMs, and *p* values were calculated to indicate statistical significance of the predictive accuracy (see Materials and Methods). We chose the *k*-folds balanced cross-validation approach, instead of a leave-one-out subject approach, because it is less susceptible to outlier participants (Vu et al., 2022).

Analyses showed that distributed, prestimulus patterns in MPFC/BA10 predict the decision to choose to think about the self with 70% accuracy, *p* = 0.01. In other words, the brain pattern a person enters in MPFC/BA10 as soon as their mind is free from external demands predicts whether they next want to think about themselves. But is it the only part of the brain that can do this? We next assessed if MPFC/BA10 predicted self-choices better than patterns from the entire brain. The whole-brain pattern during pretrial rest was able to predict the subsequent decision to choose the self with 77% accuracy, *p* = 0.001. The higher accuracy for whole-brain classification suggests additional brain areas contribute to the bias toward self-focus.

To determine which additional brain regions contribute to the bias toward self-focus, it is important to consider that MPFC/BA10 is one of multiple brain regions comprising the brain's default network and that mental phenomena can arise from distributed patterns of neural activity across interacting brain regions (Bressler and Menon, 2010; Chang et al., 2015; Kragel et al., 2018). Graph analytic methods indicate that the default network is composed of three subsystems: the core subsystem (associated with self-reflection, prospection, and autobiographical memory), dorsomedial subsystem (associated with social semantic knowledge and inferring mental states), and the medial temporal lobe subsystem (associated with episodic memory, simulation, and relational processing; Andrews-Hanna et al., 2010). As alluded to in its name, the core subsystem is the primary default network subsystem, and MPFC/BA10 is a key node. Thus, while MPFC/BA10 is the region most reliably associated with self-reflection (Lieberman et al., 2019), it is possible that distributed patterns of neural activity in the core default network subsystem, including MPFC/BA10, may contribute to the bias toward self-focus.

**Figure 3.**
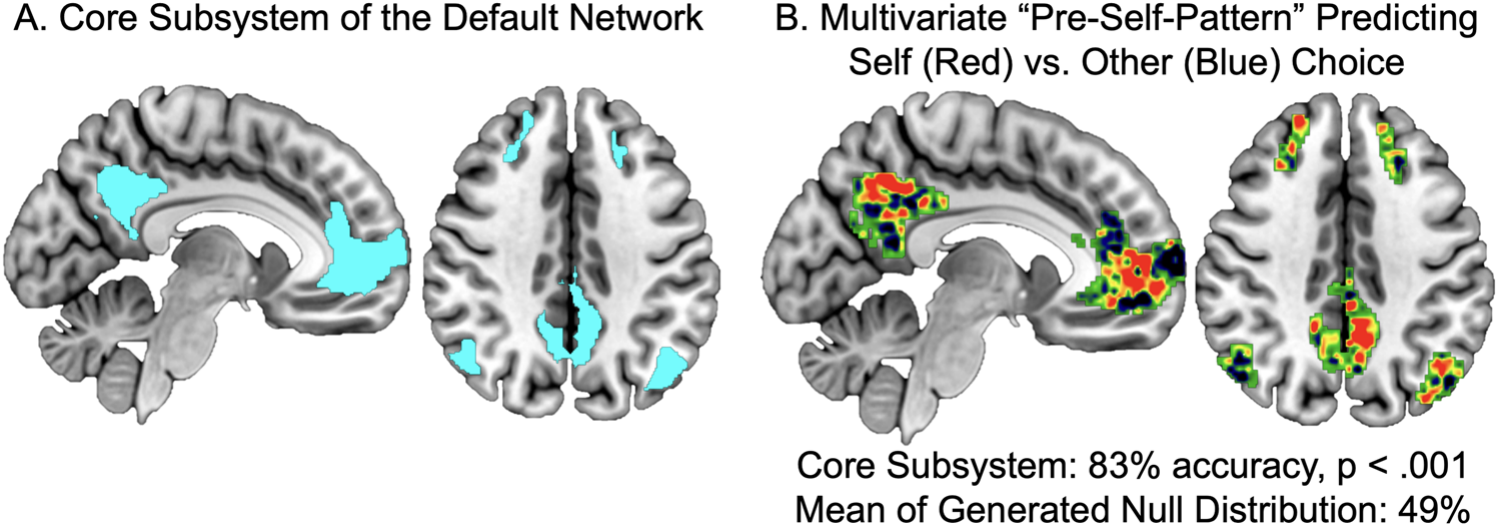
The default network's core subsystem during pretrial rest periods predicted the decision to choose to think about the self with 83% accuracy. This accuracy was significantly better (*p* < 0.001) than the average classification accuracy in the generated null distribution, which was 49%. ***A***, The default network's core subsystem. ***B***, The multivoxel pattern generated by the SVM to differentiate rest activity that proceeds self-choice rather than other-choice. Red indicates regions where activity predicts self-choice and blue indicates regions where activity predicts other-choice. The pattern shown was created with all subjects although classification accuracy was computed with a sixfolds approach.

We therefore repeated the steps for SVM classification on a whole-brain activation map masked by the three subsystems of the default network (core subsystem, dMPFC subsystem, and MTL subsystem) as mapped by Yeo et al. (2011). We found multivariate patterns in the core subsystem during pretrial rest periods predicted the decision to choose to think about the self with 83% accuracy, *p* < 0.001 (Fig. 3). The dMPFC subsystem (48% accuracy, *p* = 0.61) and MTL subsystem (59% accuracy, *p* = 0.17) both were not able to classify results above chance. These results demonstrate that distributed patterns in the default network core subsystem during pretrial rest best predict the subsequent choice to think about the self. The default network core subsystem classification accuracy is higher than the whole-brain accuracy, suggesting that the whole-brain results are largely driven by the default network core subsystem. We would expect that even though the whole-brain includes the default network core, its accuracy would be lower because of the noise created by other areas of the brain outside of the core system.

We next dug another layer deeper, to understand precisely which default network core regions besides MPFC/BA10 contribute to its classification power. We performed follow-up analyses with all of the ROIs included in Yeo's default network core subsystem: right middle temporal gyrus, right superior frontal gyrus, left middle frontal gyrus with left superior frontal gyrus, left angular gyrus, right angular gyrus with right middle temporal gyrus, posterior cingulate/precuneus (PCC), and MPFC/BA10. The only classifiers that were significant with an uncorrected threshold were the PCC (69% accuracy, *p* = 0.02) in addition to MPFC/BA10 (70% accuracy, *p* = 0.01). To determine if one region was contributing more than the other to the core subystem's predictive power, we ran an MVPA with the default network core subsystem minus MPFC/BA10 (73% accuracy, *p* = 0.004), the default network core subsystem minus the PCC (73% accuracy, *p* = 0.005), the default network core subsystem minus MPFC/BA10 and PCC (59% accuracy, *p* = 0.17), and MPFC/BA10 and PCC combined (72% accuracy, *p* = 0.006). We ran statistical tests on a total of 15 classifier models over the course of this analysis. When we correct for multiple comparisons, only the default network core subsystem (83% accuracy, *p* = 0.0001) and the whole brain (77% accuracy, *p* = 0.001) survive the corrected threshold (*p* < 0.05/15, or 0.003). These results in combination with the above suggest MPFC/BA10 and PCC equally contribute to the success of the default network core classifier accuracy, but the activation pattern of the whole default network core subsystem is needed for the best classification power. Therefore we use the core subsystem pre-self pattern—the classifier weights map generated by the multivoxel pattern classification analysis performed with all subjects' activation maps—as our primary pattern in all subsequent analyses.

#### A neural signature that predicts self-focus: evidence from the resting-state scan

##### The pre-self pattern in the default network’s core subsystem during long periods of rest predict self-reported self-focus

**Figure 4.**
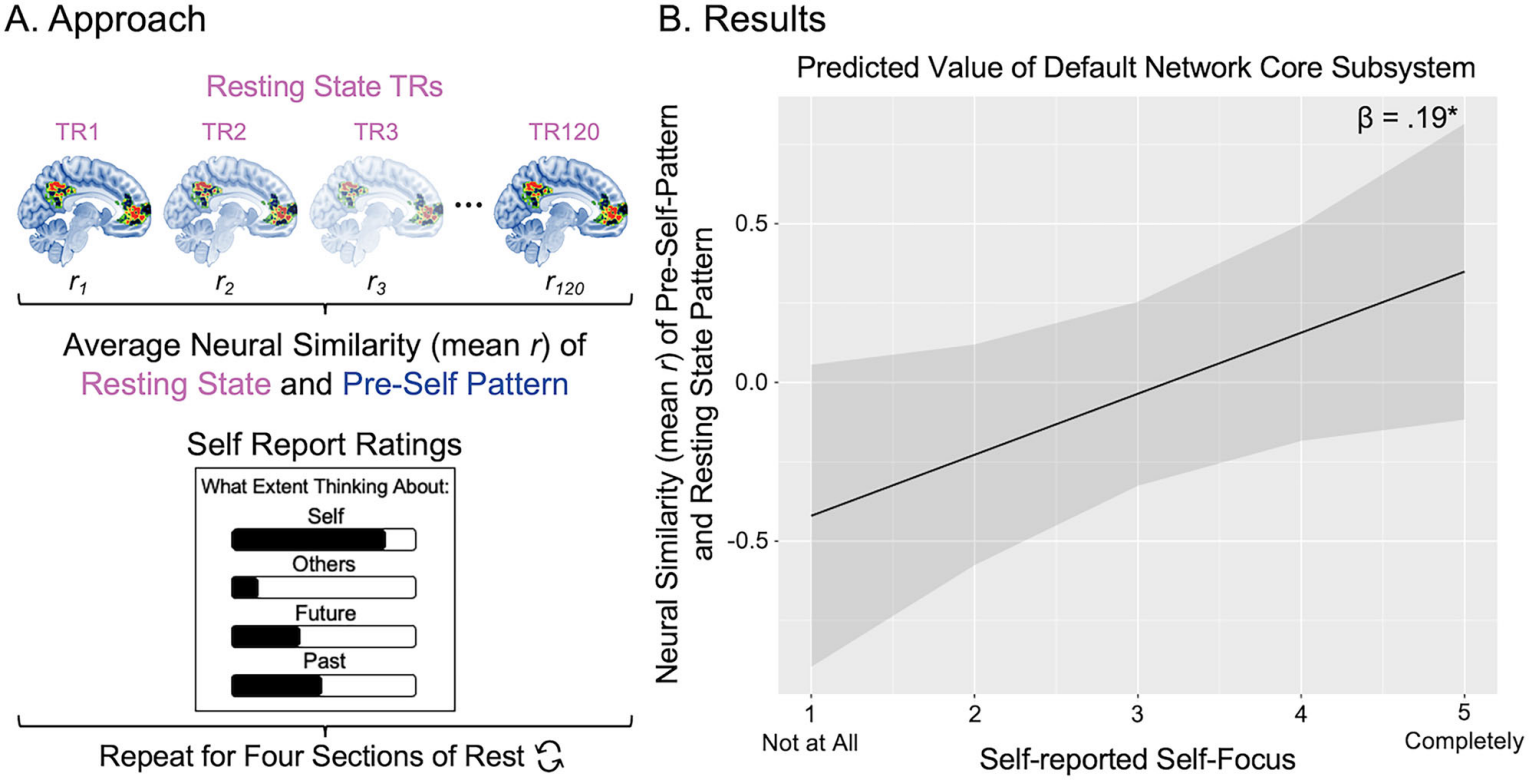
Applying the pre-self pattern to a resting-state scan with experience sampling. ***A***, Our analytic approach started with a within-subjects, TR-to-TR multivariate pattern similarity analysis, assessing the similarity between (1) the pre-self pattern and (2) subjects’ resting-state scan pattern in the default network core subsystem. Those correlation values were then averaged for the 2 min rest sections and Fisher *z*-transformed. A linear mixed model assessed how self-reported thought content (self, other, future, and past) as well as the section of rest affected the mean correlation. ***B***, In the results graph, the *y*-axis displays the normed mean correlation of the resting-state neural patterns (in the default network core) with the multivoxel classifying pre-self pattern generated (in the default network core). The *x*-axis is the self-reported self-focus rating that follows each of the 2 min sections of rest from 1 = “not at all” to 5 = “completely.” The dark gray bars reflect the confidence intervals based on standard error (± 1.96 * SE). We found that the strength of the default network core subsystem multivoxel pattern during rest was significantly related to self-reported self-focus (*β* = 0.19, standardized *β* = 0.09, *t*_(105.1)_ = 2.03, *p* = 0.045). In other words, the default network core multivariate pattern derived from short, jittered rest to predict subsequent choice to think about the self, is also able to decode self-focused thought during resting state.

So far, we have identified a pre-self pattern which predicts self-focused behavior in a forced choice task. Our next goal was to determine if the pre-self pattern generalizes to predict self-focus in another context—a resting-state scan. In the beginning of our experiment, participants completed an 8 min resting-state scan and every 2 min completed a series of self-reports. They were asked to rate, on a 1 to 5 scale, how much they are thinking about themselves, others, the past, and the future (Fig. 1*B*). Here, we asked: does the presence of the pre-self pattern preferentially predict self-reported self-focus? We performed an instatement analysis—a within-subjects, TR-to-TR multivariate pattern similarity analysis—assessing the similarity between (1) the pre-self pattern and (2) subjects' resting-state scan pattern in the default network core subsystem. Specifically, each TR of the rest data was masked by the Yeo (2011) default network core subsystem and correlated with the default network core subsystem pre-self pattern (Fig. 4*A* provides a visualization of the approach). Correlation values were then averaged for the 2 min rest sections and Fisher *z*-transformed. A linear mixed model assessed how self-reported thought content (self, other, future, and past) as well as the section of rest affected the mean correlation. To ensure that the regressors were not introducing collinearity to the model we ran tolerance and variance inflation factors (VIFs). Results indicated that collinearity is not an issue in our model: all tolerance values were over 75% (Self, 82; Other, 90; Future, 77; Past, 88; and Section, 93), and all VIF results were well below the threshold of 5 and approaching the baseline of 1 which demonstrates no collinearity (Kutner et al., 2005; Self, 1.2; Other, 1.1; Future, 1.3; Past, 1.1; and Section, 1.1).

A stronger presence of the pre-self pattern corresponded with greater self-reported self-focused thought (*β* = 0.19, standardized *β* = 0.09, *t*_(105.1)_ = 2.03, *p* = 0.045; Fig. 4*B*) but not other-focused thought (*β* = 0.07, standardized *β* = 0.08, *t*_(104.1)_ = 0.85, *p* = 0.40), future-focused thought (*β* = −0.01, standardized *β* = 0.08, *t*_(92.6)_ = −0.10, *p* = 0.92), past-focused thought (*β* = 0.05, standardized *β* = 0.07, *t*_(86.8)_ = 0.70, *p* = 0.49), or the section of the resting-state scan (*β* = 0.06, standardized *β* = 0.06, *t*_(80.1)_ = 0.91, *p* = 0.37). Follow-up analyses with the whole brain, dmPFC and MTL subsystems, as well as MPFC/BA10 and PCC examined individually, did not produce significant results (*β*'s < 0.14, *p's* > 0.10). Thus, multivariate patterns in the default network core subsystem as a whole—derived from short, jittered rest to predict subsequent choice to think about the self—is also able to predict self-reported, self-focused thought during a resting-state scan.

##### The pre-self pattern temporally predicts the active self-reflection neural pattern during a resting-state scan

We have shown the pre-self pattern predicts behavioral markers of self-focus: self-focused decisions and self-reported self-focus. Could the pre-self pattern even predict a neural marker of self-focus that participants do not actively report on? To answer this question, we used two multivariate patterns—(1) the pre-self pattern and (2) a separate multivariate pattern that reflects active self-focus. We examined when these two patterns occurred during the subjects' resting-state scan, assessing whether the pre-self pattern temporally predicted the subsequent occurrence of the active self-focus pattern.

To generate the neural pattern of active self-reflection, we computed, for each subject, a multivariate pattern that reflected their active self-reflection in our final fMRI task, in which they answered questions about themselves such as “am I an ambitious student?” (Fig. 1*C*; see Study 1 Fig. S1 for a visualization of the active self-reflection pattern). We then used instatement analysis on the resting-state scan to test whether the presence of the pre-self pattern precedes the presence of this active self-reflection pattern in the core default network subsystem. We restricted our analyses to the core subsystem because of the accumulating evidence so far that distributed patterns in this network meaningfully predict self-focus.

**Figure 5.**
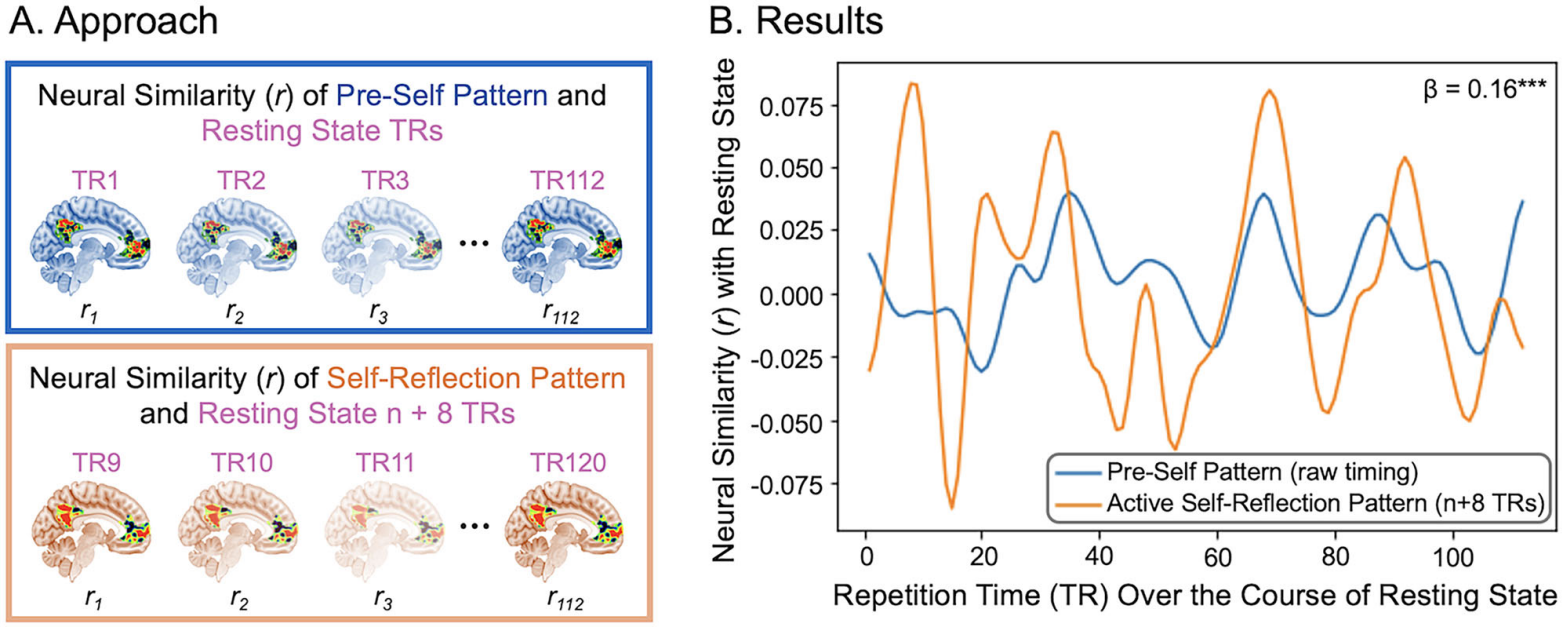
Relationship between pre-self pattern and active self-reflection pattern over the resting-state scan (***A***) depicts the approach using an 8 TR delay: the pre-self pattern (blue) was correlated with each TR (i.e., 1 s) of the resting-state scan and the active self-reflection pattern (orange) was also correlated with each TR of the resting-state scan. Linear mixed models (Bates et al., 2015) assessed if pre-self pattern correlation strength predicted active self-reflection pattern correlation strength 0–20 TRs later (here 8 TRs later). This allowed us to assess whether the presence of the pre-self pattern temporally predicted the presence of the active self-reflection pattern at multiple lag times. ***B***, The results for a single subject using an 8 TR delay, demonstrating that the presence of the pre-self pattern (blue) predicts the presence of the active self-reflection pattern (orange) 8 s later. Note that the active self-reflection pattern strength visualized in orange has been shifted in time (*n* + 8 s) to help visualize its relationship with the pre-self pattern.

We performed an instatement analysis, taking each participant’s multivariate self-reflection pattern in the core subsystem from the final fMRI task and assessing its neural pattern similarity with each second (i.e., TR) of the resting-state scan. We then assessed if resting-state neural pattern similarity to the pre-self pattern temporally predicted neural pattern similarity to the self-reflection pattern at different timing delays. Specifically, linear mixed models (Bates et al., 2015) assessed if pre-self pattern correlation strength predicted active self-reflection pattern correlation strength 0–20 TRs later (see Fig. 5*A* for a visualization of the approach). We chose to use this data-driven approach with multiple TR lag times to avoid arbitrarily selecting a time delay as well as to better understand the dynamic temporal relationship between the presence of the “pre-self” pattern and active self-reflection pattern. Analyses are performed on Fisher *z*-transformed correlation values.

It is noteworthy that the pre-self pattern and each subjects' self-reflection pattern demonstrated small correlations (−0.12 < *r*'s < 0.10, mean *r* = −0.03, SD *r* = 0.05). We therefore ran the linear mixed models with 0–20 TR delays: (1) with the strength (i.e., correlation) of the pre-self pattern predicting the strength of the active self-reflection pattern X TRs later and (2) with the strength (i.e., correlation) of the active self-reflection pattern predicting the strength of the pre-self pattern X TRs later. To account for the number of statistical tests performed (41), we used a Bonferroni-corrected significance value of *p* < 0.00122 (i.e., 0.05 × 41).

Our first observation further indicated that the pre-self pattern and active self-reflection patterns are not redundant with one another and instead are distinct brain patterns. The presence of the pre-self pattern and the presence of an active self-reflection pattern were negatively correlated when we used no TR delay (*β* = −0.16, standardized *β* = 0.03, *t*_(15340)_ = −4.70, *p* < 0.00001) as well as when there was a 1 or 2 TR delay of the pre-self pattern predicting the active self-reflection pattern (1TR: *β* = −2.47, standardized *β* = 0.04, *t*_(15210)_ = −7.16, *p* < 0.00001; 2TR: *β* = −1.27, standardized *β* = 0.04, *t*_(15080)_ = −3.65, *p* = 0.0003). These results demonstrate that our pre-self pattern is capturing neural activity that is distinct and different from active self-reflection. The negative correlations suggest that when someone is more strongly in the pre-self pattern “state,” they are less likely to be in the active self-reflection “state,” pointing to their distinctness.

Our second observation was that there is a temporal relationship between the pre-self pattern and active self-reflection pattern. The strength of the pre-self pattern significantly predicted the strength of the active self-reflection pattern 8 TRs later (*β* = 0.16, standardized *β* = 0.04, *t*_(14310)_ = 4.55, *p* < 0.00001). Figure 5*B* depicts this finding for one example participant. This temporal relationship also appeared 11 TRs later (*β* = 0.16, standardized *β* = 0.04, *t*_(13930)_ = 4.44, *p* = 0.00001), 13 TRs later (*β* = 0.14, standardized *β* = 0.04, *t*_(13670)_ = 3.94, *p* = 0.00008), and 14 TRs later (*β* = 0.15, standardized *β* = 0.04, *t*_(13540)_ = 4.08, *p* = 0.00005). In contrast, the active self-reflection patterns did not significantly predict a stronger presence of the pre-self pattern with any time delay (*t*’s −2.37–2.55, *p*’s 0.01–0.96), apart for the 1 and 2 TR delays showing a negative relationship that are reported in the above paragraph. See Study 1 Table S1 for a full list of the results for each TR lag. These results suggest multivariate patterns in the default network core subsystem—derived from short, jittered rest to predict subsequent choices to think about the self—are also able to temporally predict neural indices of active self-reflection during a resting-state scan, even when the neural indices of active self-reflection are clearly distinct from the pre-self pattern by virtue of them being negatively correlated at the same moment in time.

We also capitalized on the active self-reflection pattern to test whether the relationship between the pre-self pattern and self-reported self-focus (reported in the section above) is independent of a relationship between the active self-reflection pattern and self-reported self-focus. A linear mixed model that included both the pre-self pattern and the active self-reflection pattern as independent predictors of self-reported self-focus showed that there was still a significant relationship between pre-self pattern instatement and self-reported self-focus (*β* = 0.20, standardized *β* = 0.10, *t*_(102.4)_ = 2.08, *p* = 0.041). Indeed, when we looked at variance inflation factors, VIF results were well below the threshold of 5 and approaching the baseline of 1 which demonstrates no collinearity (Kutner et al., 2005; “pre-self” pattern = 1.01 and self-reflection pattern = 1.02). This further suggests that the pre-self pattern is not redundant with active self-reflection.

### Study 2: testing implications for internalizing and out-of-sample testing

#### A neural signature that predicts self-focus: evidence from the Human Connectome Project

##### In the Human Connectome Project dataset, high internalizers move in and out of the pre-self pattern similarly over time

We arbitrated between two ways in which instating the pre-self pattern during rest may relate to internalizing. The first possibility is that an overall greater degree of pre-self pattern instatement predicts internalizing, given that individuals higher on this trait dimension report an overall greater amount of self-focus. To test the first possibility, for each participant, we computed the average strength (i.e., correlation) in the core subsystem between the pre-self pattern and their rest pattern across all TRs of the resting-state scan. We then tested if internalizing scores predicted the average amount of pre-self pattern instatement during rest. Internalizing scores were not significantly related to the overall amount of pre-self pattern instatement (*β* = −0.00001, standardized *β* = −0.02, *t*_(1085)_ = −0.62, *p* = 0.54). Put simply, high internalizers do not instate the pre-self pattern to a greater degree than low internalizers.

**Figure 6.**
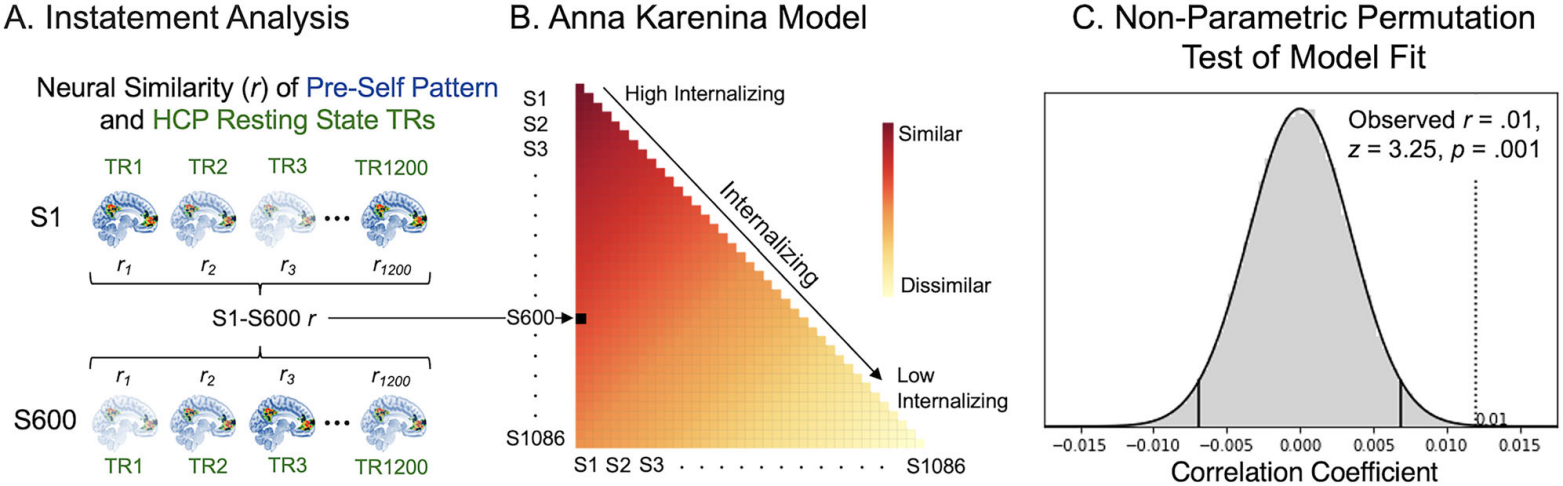
Intersubject representation similarity analysis (IS-RSA) with Anna Karenina model: high internalizations have a similar pre-self pattern time course during a resting-state scan. The Anna Karenina model testing whether high internalizers move in and out of the pre-self pattern similarly over the course of a resting-state scan. ***A***, Similarity in the timing of the pre-self pattern strength was computed for all pairs of Human Connectome Project participants and compared with (***B***) the theoretical model that high internalizers show a similar time course to one another while low internalizers show idiosyncratic time courses. ***C***, A nonparametric, Mantel permutation test (see Materials and Methods) showed the internalizing Anna Karenina model was significant.

**Figure 7.**
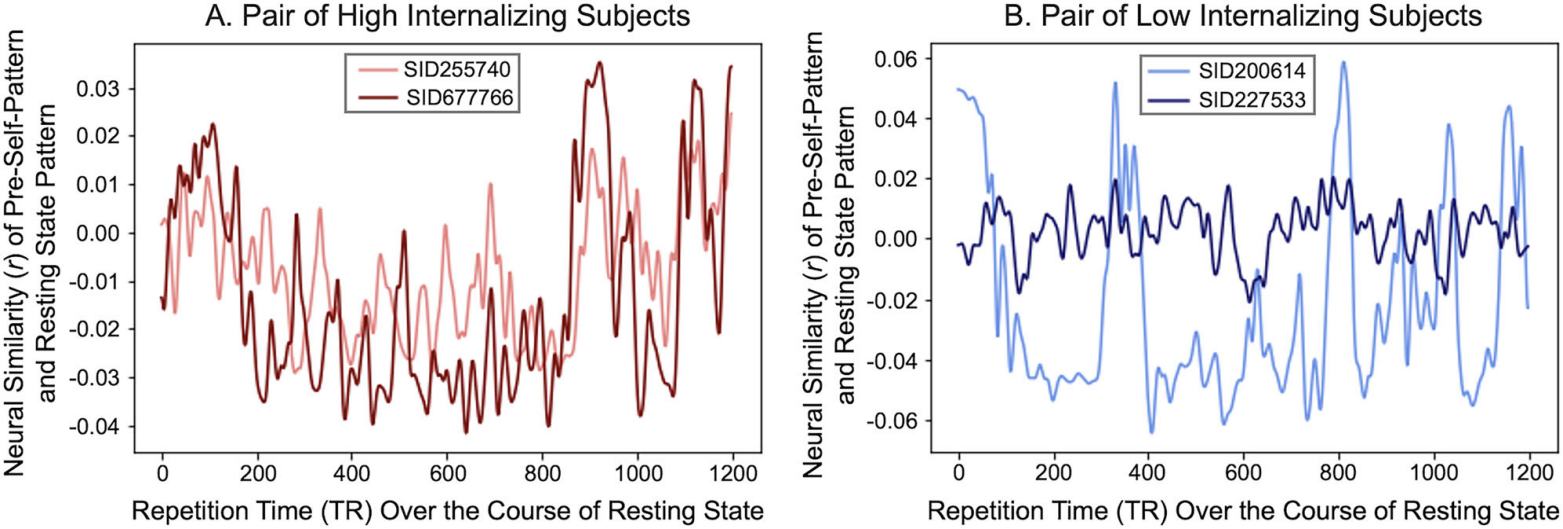
Intersubject representational similarity analysis (IS-RSA): Example subjects' pre-self pattern instatement time course for our IS-RSA. We compared the time course of individuals' pre-self pattern instatement strength with an Anna Karenina model of internalizing such that the higher a pair's internalizing rank, the higher their similarity in pre-self pattern instatement timing, whereas the lower their internalizing rank, the more idiosyncratic their timing. ***A*** shows two high-internalizing subjects (90th percentile) pre-self pattern instatement time course while ***B*** shows two low-internalizing subjects (10th percentile) time course.

Next, we tested the second possibility: individuals higher on the internalizing trait dimension move in and out of the pre-self pattern similarly over the course of rest. This possibility is consistent with recent research showing that individuals with similar trait characteristics show similar time courses of neural responding (Finn et al., 2018), including over the course of resting-state scans (Iyer et al., 2024). To test this possibility, we performed an Anna Karenina IS-RSA, which can test whether individuals who score high on a trait dimension show similar neural responses, whereas individuals who score low on the same trait dimension are neither similar to other low nor high scorers (i.e., there is no systematic structure to low scorers' neural responding; Fig. 6). We chose this model because we did not expect any particular timing with respect to when low internalizers instate their pre-self pattern. In contrast, we reasoned high internalizers may instate their pre-self pattern in systematic ways (and hence similarly) over time. The Anna Karenina model was significant (*r* = 0.01, *z* = 3.25, *p* = 0.001, Mantel permutation test; Fig. 6*C*). In other words, people high on internalizing, a clinical variable highly related to maladaptive self-focus, move in and out of the pre-self pattern similarly over time throughout a resting-state scan. For visualization purposes, Figure 7 shows pre-self pattern instatement time courses from four Human Connectome participants: two who score high (left panel) and two who score low (right panel) on internalizing.

The internalizing measure used in the Human Connectome Project includes three subscales: (1) Anxiety and Depression, (2) Withdrawal, and (3) Somatic Symptoms. Importantly, the first two dimensions relate to psychological self-focus more so than a focus on one’s bodily sensation. As a follow-up, we performed IS-RSA analyses, separately for each of three of the subscales within the internalizing measure. Both the Anxiety and Depression subscale and the Withdrawal subscale showed a significant Anna Karenina effect (Anxiety and Depression: *r* = 0.014, *z* *=* 4.79, *p* < 0.00001; Withdrawal: *r* = 0.01, *z* *=* 2.86, *p* = 0.004). Somatic Complaints, however, did not show a significant effect (*r* = 0.0001, *z* *=* 0.02, *p* = 0.98).

Supplementary Materials (Text S2) detail follow-up analyses that further confirm that high internalizers similarly instate the pre-self pattern over time during rest. Briefly here, the results could not be attributed to (1) similar pre-self pattern instatement time courses for similar levels of internalizing scores across the sample, rather than high internalizing scores specifically, or (2) high internalizers showing different degrees of variability in their pre-self pattern instatement during rest. Collectively, the Human Connectome Project findings suggest that people high on internalizing, particularly anxiety, depression, and withdrawal, show similar timing to the presence of the pre-self pattern during a resting-state scan, suggesting there may be a systematic, temporal structure to their spontaneous, self-focused thought.

## Discussion

Although self-focus is a ubiquitous part of human psychology, the processes generating interest in ourselves remain undercharacterized. We identified a brain pattern that when entered during brief mental breaks predicts next focusing on oneself. In Study 1, a multivariate neural pattern in the default network's core subsystem, which includes MPFC/BA10, could decode the subsequent decision to focus on the self with high accuracy. The pre-self pattern in the default network's core subsystem also predicts self-reported self-focus during a resting-state scan. It is even capable of temporally predicting the presence of a neural pattern capturing active self-reflection during a resting-state scan. In Study 2, we applied the pre-self pattern to data from the Human Connectome Project and found our pre-self pattern significantly relates to participants' internalizing scores. Individuals with high internalizing scores move in and out of the pre-self pattern in similar ways during a resting-state scan, whereas individuals with low internalizing scores showed no similar timing to their pre-self pattern. This result offers a unique way to think about how internalizing relates to self-focus—there may be a systematic timing to high internalizers' spontaneous, self-focused thought in the core default network.

What is going on—in terms of a psychological process—in participants' brains when we observe the pre-self pattern? While the pre-self pattern modestly correlates with a pattern reflecting active self-reflection (from a task in which participants must reflect on themselves), it is not redundant with the active self-reflection pattern. Rather, in any given moment, the presence of the pre-self pattern negatively correlates with the presence of the active self-reflection pattern, yet it can temporally predict the later presence of the active self-reflection pattern. The pre-self pattern and active self-reflection patterns also explain unique variance in participants’ self-reported self-focus. Together, the data suggests whatever psychological process may be captured by our pre-self pattern, it appears distinct from active self-reflection.

There may be at least three possible psychological phenomena captured by our pre-self pattern. First, the pre-self pattern may signify a “loading” of the self, a coming online of a self-schema through which to view and interact with the world. Consistent with this view, some posit that we constantly exist in a framework of the self, as we understand our world through our own experiences and embodied sense of self, which we constantly exist in (Christoff et al., 2011). The second possibility is related to the first: the pre-self pattern could reflect the persistent cognitive accessibility of the self (Bruner, 1957; Libet et al., 1983; den Daas et al., 2013). Cognitive category accessibility was first proposed by Bruner (1957) and suggests that the attentional framework we are in directly influences what we subsequently perceive or think about. Thus, the pre-self pattern may reflect an attentional state that facilitates quick access to self-knowledge. Third, it is possible the pre-self pattern represents a motivational state. Participants may be experiencing a temptation or impulse to think about themselves. This may be an urge that they could resist or succumb to, perhaps with significant implications for mental health, specifically rumination. Though we cannot be certain which of these possibilities the pre-self pattern captures, its observation fits with predictive coding accounts of brain function, which broadly suggest endogenous, default brain patterns predict subsequent perception and cognition (Hutchinson and Barrett, 2019).

Another important question to address about the pre-self pattern is whether it captures conscious preparation or deliberation to think about the self versus a more implicit process. Prior work has observed that nonconscious prestimulus activity can predict subsequent decisions (Soon et al., 2008, 2013; Huang et al., 2014). Previous fMRI and intracranial recording research shows neural activity in MPFC and PCC—the two regions in our data with the strongest classification power to predict subsequent self-focused thought—predicts subsequent decisions to press a button or perform a math task 2–7 s before participants report a conscious choice (Soon et al., 2008, 2013; Fried et al., 2011). Relatedly, EEG research on the “readiness potential,” a build-up of activity in the prefrontal cortex prior to the conscious decision to move (Libet et al., 1983; Schurger et al., 2021), led researchers to theorize that when spontaneous neural activity passes a threshold or coincides with a signal from a different brain region, it can nudge a participant into motion without prior, conscious deliberation (Schurger et al., 2012, 2021; Murakami et al., 2017). This fits with our current findings, as our self- versus other-focused decision task allows us to capture a (perhaps preconscious) build-up of neural activity before the choice to be self-focused.

Social psychological theories argue humans are interested in themselves to meet both an epistemic goal to understand their reality and a belonging goal to feel connected to social groups (Leary, 2007; Kwang and Swann, 2010). Our results offer a new twist on these ideas—a brain pattern we enter reflexively, even in just a few second mental break, may help ensure we default toward trying to meet these goals. Future work can test this directly, for example, by examining (1) whether engaging the pre-self pattern during brief mental breaks guides decisions that help meet epistemic and belonging goals and (2) whether manipulating the need to achieve these goals increases instatement of the pre-self pattern during brief mental breaks.

The pre-self pattern may also update models of cognitive phenomena related to the self. The “self-reference effect” refers to the bias toward remembering self-relevant information. The self-reference effect is thought to occur because people have highly elaborate and organized self-schemas, which facilitates higher quality processing of self-relevant content during encoding (Symons and Johnson, 1997). Our work suggests another possibility. Because the pre-self pattern emerges by default during momentary mental breaks, having it active just prior to encoding new stimuli may make stimuli that are related to the self more efficiently incorporated into self-knowledge. This idea fits with the concept of “preplay” from animal research; neurons that are already active in a rodent during brief rest before completing a novel maze direct behavior and subsequent memory of their maze trajectory (Buhry et al., 2011). Future work can test whether spontaneously instating the pre-self pattern during brief mental breaks shapes learning and memory for self-related information appearing moments later.

The pre-self pattern may be a useful decoding tool in mental health research. Among Human Connectome Project participants, similarly moving in and out of the pre-self pattern during a resting-state scan corresponded with internalizing symptoms, particularly depression, anxiety, and withdrawal. Rumination—repetitive and recurrent negative thinking about oneself (Watkins, 2008)—is thought to play a key role in internalizing disorders (du Pont et al., 2018; Watkins and Roberts, 2020; Baez et al., 2023). Yet, the neural mechanism that explains why and how rumination spontaneously occurs is not fully understood, although past work has associated the default network with depression and anxiety (Sheline et al., 2009; Coutinho et al., 2016). The pre-self pattern may offer key insight into the basic mechanisms underlying rumination. The tendency to systematically move in and out of the pre-self pattern may bias high internalizers toward the repetitive, negative thoughts characteristic of rumination. Moreover, the pre-self pattern could be applied to patient populations and individuals at risk for developing internalizing disorders to help predict maladaptive self-focus. Down the line, applying the pre-self pattern with neurofeedback methods could even offset ruminative self-focus.

It is noteworthy that high internalizing was associated with similar pre-self timing during a resting-state scan, given that during rest there are no shared external stimuli to drive synchrony. Synchrony in the absence of a shared stimulus likely reflects that trait internalizing corresponds with stable tendencies in the way self-focused thought endogenously emerges while mind wandering. The data collection paradigm also facilitated the ability to capture trait-level individual differences in mind wandering because the resting-state scan occurs at the very beginning of the scanning session and uses a very quick sampling rate (0.72 s TRs). Thus, participants begin mind wandering at a very similar point in the experiment, and small shifts in data collection start times should not highly influence the imaging data that was collected. That said, at this stage it is still unclear what synchrony in pre-self pattern instatement reflects among high internalizers. Indeed, while the results are statistically significant, the effect sizes are relatively small. This could be because that, while on average and across so many participants, we are sampling high internalizers at similar moments of their mind wandering, some are offset because resting-state scan analyses are not time locked to a stimulus. This would suggest that our effect size is an underestimate of the true effect due to noise. Another possibility is that the IS-RSA results are detecting some unique structure to high internalizers' pre-self pattern time courses, such as less variance in its state transitions. Future work is needed to arbitrate between these and other related possibilities. There is also a growing appreciation that individual differences in brain function may be best captured by using ambiguous, naturalistic stimuli (Finn, 2021; Finn and Bandettini, 2021), because they strike a nice balance between the ability for individual differences to emerge while not compromising experimental control and interpretability of temporal patterns. Future work could use naturalistic stimuli designed to be self-relevant to high but not low internalizers and assess whether there are greater intersubject correlations of pre-self pattern timing among high versus low internalizers.

Self-focus is a pervasive human phenomenon that plays a vital role in our lives—from meeting our personal needs to understanding our social standing. However, in its most pernicious forms, self-focus is a risk and maintenance factor for internalizing disorders such as depression and anxiety. We document a neural signature—the pre-self pattern—that biases people toward self-focus. The pre-self pattern predicts self-focused decisions, subjective self-focus during rest, and the presence of active self-reflection neural patterns a few seconds later. Moreover, the timing of the pre-self pattern during rest predicted internalizing scores Human Connectome Project participants. The pre-self pattern gets us closer to understanding why humans cannot help but focus on themselves, as well as how this process is altered in mental health conditions.

## Data Availability

Data from our experiment can be found here, https://osf.io/8gefx/, and Human Connectome Data that we analyzed can be found here, https://db.humanconnectome.org/
